# Changes in Morphological and Elastic Properties of Patellar Tendon in Athletes with Unilateral Patellar Tendinopathy and Their Relationships with Pain and Functional Disability

**DOI:** 10.1371/journal.pone.0108337

**Published:** 2014-10-10

**Authors:** Zhi Jie Zhang, Gabriel Yin-fat Ng, Wai Chun Lee, Siu Ngor Fu

**Affiliations:** 1 Department of Rehabilitation Sciences, The Hong Kong Polytechnic University, Hung Hom, Kowloon, Hong Kong; 2 Guangdong-Hongkong Joint Sports Rehabilitation and Research Center, Guangdong Provincial Work Injury Rehabilitation Hospital, Guangzhou, China; The University of Queensland, Australia

## Abstract

**Background:**

Patellar tendinopathy (PT) is one of the most common knee disorders among athletes. Changes in morphology and elasticity of the painful tendon and how these relate to the self-perceived pain and dysfunction remain unclear.

**Objectives:**

To compare the morphology and elastic properties of patellar tendons between athlete with and without unilateral PT and to examine its association with self-perceived pain and dysfunction.

**Methods:**

In this cross-sectional study, 33 male athletes (20 healthy and 13 with unilateral PT) were enrolled. The morphology and elastic properties of the patellar tendon were assessed by the grey and elastography mode of supersonic shear imaging (SSI) technique while the intensity of pressure pain, self-perceived pain and dysfunction were quantified with a 10-lb force to the most painful site and the Victorian Institute of Sport Assessment-patella (VISA-P) questionnaire, respectively.

**Results:**

In athletes with unilateral PT, the painful tendons had higher shear elastic modulus (SEM) and larger tendon than the non-painful side (*p*<0.05) or the dominant side of the healthy athletes (*p*<0.05). Significant correlations were found between tendon SEM ratio (SEM of painful over non-painful tendon) and the intensity of pressure pain (*rho*  = 0.62; *p* = 0.024), VISA-P scores (*rho*  = −0.61; *p* = 0.026), and the sub-scores of the VISA-P scores on going down stairs, lunge, single leg hopping and squatting (*rho* ranged from −0.63 to −0.67; *p*<0.05).

**Conclusions:**

Athletes with unilateral PT had stiffer and larger tendon on the painful side than the non-painful side and the dominant side of healthy athletes. No significant differences on the patellar tendon morphology and elastic properties were detected between the dominant and non-dominant knees of the healthy control. The ratio of the SEM of painful to non-painful sides was associated with pain and dysfunction among athletes with unilateral PT.

## Introduction

Patellar tendinopathy (PT) is a common and often chronic knee disorder among competitive athletes [1]. Its prevalence has been reported to be as high as 30% to 45% in athletes involved in jumping sports [2]. Subjects with PT are characterized with localized pain at the proximal patellar tendon associated with jumping and squatting activities that load the tendon [3]. Since the primary function of tendon is to transmit tensile loading, any change in its morphology and elastic properties may affect its function during normal activities.

Tendinopathy results in disruption and disorganization of the tendon fibers [4], along with increases in tendon thickness [3], [5] and cross-sectional area (CSA) of the structure affected [6]. Based on ultrasound imaging, Cook et al. [7] observed hypoechoic changes in human tendons with tendinopathy. The authors thereby recommended the use of ultrasonography in addition to clinical examination to confirm the diagnosis of PT. Alteration in the elastic properties of patellar tendon, however, have not been adequately described. In individuals with PT, the tendon was found to be more elastic in one study [8] but no difference was reported in another 2 studies [9]–[10] when compared with controls. In those studies, tendon stiffness was assessed using ultrasound imaging with dynamometry. This technique measures the elastic properties of the whole tendon during ramped maximum voluntary isometric contraction. Clinically, pathological lesions in PT typically occur at about 5 mm from the apex of the patella [3], [11], therefore site-specific evaluation at the pathological region might shed light on the changes on tissue elastic properties associated with tendinopathy.

Recently, strain imaging has been used to assess regional tendon elastic properties [12]–[13]. A compressive force is applied either manually or by the emission of low radiofrequency impulses via an ultrasound probe to the tendon surface causing tendon displacement. Tissue elasticity is graded as either soft, intermediate or hard and expressed in colour-coded images (elastogram) [12]. Based on this technique, the common extensor tendon was found softer in subjects with lateral epicondylitis [13] but harder among those with Achilles tendinopathy [12] than healthy controls. To date, regional-specific evaluation on tendon elasticity associated with tendinopathy is scarce and findings are conflicting. In addition, the strain imaging technique provides qualitative but not quantitative information of tissue elasticity [14]–[15]. Thus, the magnitude of changes could not be quantified.

The supersonic shear imaging (SSI) technique provides quantitative values of tendon elastic properties at a selected area of interest [16]–[20]. It relies on measuring the speed of propagation of shear waves generated by acoustic radiation force and to estimate the shear elastic modulus of soft tissues [20]. Our recent findings indicated that the patellar tendon shear elastic modulus measured using SSI has good intra- and inter-rater reliability and is correlated with the Young's modulus of the tissue [17]. Based on this technique, decreases in tendon elastic modulus in acute ruptured Achilles tendon in human subjects [18] and in partial tendon tears in a porcine model [19] were reported. These studies provide the evidence base on the feasibility of using SSI for measuring tendon elastic properties. However, these findings cannot be generalized to tendon with tendinopathy because this condition is a degenerative process [21]. In addition to interruption of tendon fibrils [22], changes in fiber type [23] and increases in collagen cross-link concentration [24] were detected in tendon with tendinopathy. Based on the SSI technique, quantitative regional-specific tendon elasticity could be measured and compared between subjects with and without patellar tendinopathy. Such information could increase our understanding in regional changes on tissue elastic properties as well as the magnitude of changes.

There is also a question of how the changes in tendon morphological and/or elastic properties in individuals with PT relate to their perceived pain and dysfunctions. Increased tendon thickness has been reported to be associated with greater pain among athletes with PT [25]. To date, the relationship between elastic properties of tendon and self-perceived pain in individuals with PT has not been investigated. In view that pain and decrease in functional strength in the tendons could mean an end to the athletic career of a sportsman, it is therefore important to find out how changes in tendon morphology and mechanical properties are related to the disability and dysfunctions in people with PT, so that appropriate remedial measures can be developed.

The objectives of this study were to 1) compare the elastic modulus of patellar tendon between dominant and non-dominant sides among healthy subjects; 2) compare tendon on the painful and non-painful side of the same subject and also with healthy control subjects and; 3) determine whether changes in tendon shear elastic modulus were related to pain and dysfunction.

## Materials and Methods

### Ethics statement

This study was approved by the Human Subject Ethics Subcommittee of the administrating institution. The experimental procedures were conducted in accordance with the Declaration of Helsinki. The procedures of the study were fully explained to the participants and they provided their informed written consent before testing.

### Study population

Subjects were recruited from the volleyball, basketball and handball teams of local universities and the community. Only males were recruited, because PT is more prevalent in male than female athletes [2]. The inclusion criteria were as follows: 1) between 18 and 35 years of age; 2) had unilateral pain in the inferior pole of patella or the proximal part of patellar tendon; 3) pain duration ≥3 months; 4) maximum intensity of pain in the previous week ≥3 using a visual analogue scale (VAS) with 0 as no pain and 10 as the worst pain; 5) VISA score ≤80 [26]; 6) no history of corticosteroid injection or surgery to the lower limb. All recruited subjects were physically assessed by an experienced physical therapist (WCL) who has 13 years of clinical experience and then ultrasonography examination was conducted by another physical therapist (ZJZ) who has 3 years of experience in ultrasound scanning. The subject was diagnosed as having PT based on the clinical examination and ultrasonography findings that included the following: 1) local tenderness in the inferior pole of patella, or the proximal part of patellar tendon; 2) pain aggravation during single leg squatting and jumping [27]; 3) thickening of proximal part of patellar tendon with area of hypoechoic signals [6]. Twenty healthy athletes, with similar age and training hours but without clinical symptoms or abnormal ultrasound-based images of the patellar tendon, were recruited as the controls for this study.

All subjects filled in a form recording their age, weight, height and training duration per week. Subjects with PT completed the Victorian Institute of Sport Assessment-patella (VISA-P) questionnaire. The leg of dominance was determined by asking the subject to kick a ball [28].

### Ultrasound Examination

An Aixplorer ultrasound unit (Supersonic Imaging, Aix-en-Provence, France) in conjunction with a 50-mm linear-array transducer at 4–15 MHz frequency was used in this study [20]. B-mode was used to measure the tendon thickness and CSA. Shearwave mode was used to measure the shear elastic modulus of the patellar tendon at its proximal part. This location was selected because the pathological changes with diagnostic imaging for PT are most commonly found in the proximal part of the patella at about 5 mm distal to the apex of the patella [6]. The musculoskeletal acquisition mode was used to measure the elastic modulus of patella tendon with the temporal averaging (persistence) and spatial smoothing set to medium and 6, respectively. The elastic modulus measurements were taken at 1 Hz.

Each participant was examined in supine lying with 30° of knee flexion [29]. The knee was supported on a firm towel and a custom-made ankle stabilizer to keep the leg in neutral alignment on the coronal and transverse planes. Prior to testing, the subject was allowed to have 5 minutes of rest in a comfortable position in order to unload the tension on the patellar tendon [13]. The room temperature was controlled at 25°C.

Measurement of patellar tendon thickness and CSA

The thickness and CSA in the inferior pole of patella were measured by grey scale mode (B-mode) of the Aixplorer ultrasound unit. The inferior pole of patella was identified by palpation of the examiner. The transducer was lightly located at the inferior pole of the patella and the transducer was placed longitudinally on the patellar tendon. B- mode was activated to capture the image of the patellar tendon and stored for off-line measurements. The transducer was then turned by 90^o^ so that a transverse view of the proximal insertion of the patellar tendon could be captured and stored for off-line analysis. Three images were obtained for measuring the thickness and CSA [30]. Both knees were evaluated for all the subjects.

**Figure 1:**
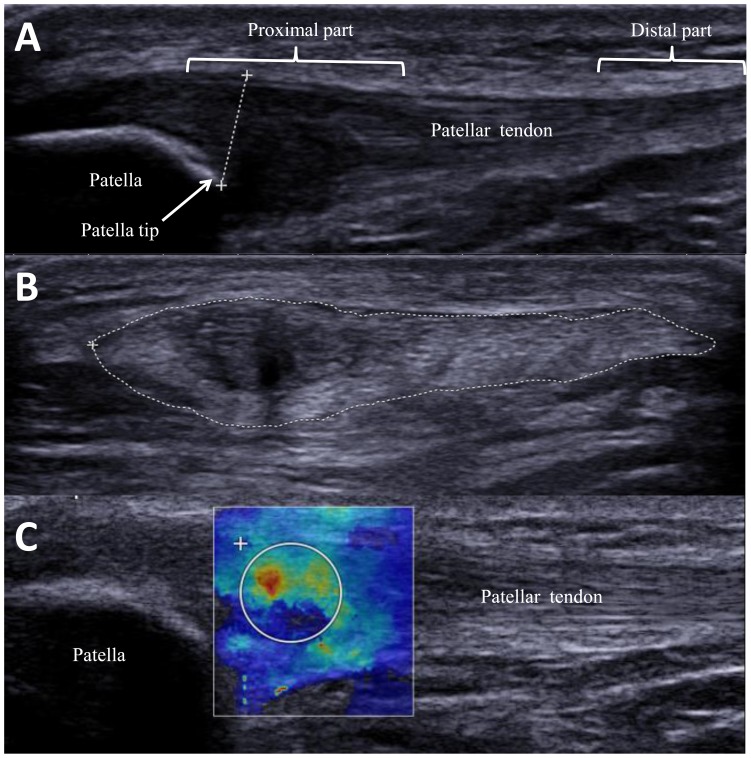
Sonography images of the patellar tendon (A) Thickness of the patellar tendon (dotted line) was measured from the superior border of the patellar tendon to the tip of the patellar. (B) Cross-sectional area of the patellar tendon was measured by tracing the outer margin of the patellar tendon (dotted circle) (C) Shear elastic modulus of the patellar tendon was quantified by the elastography. The white circle delineates the area of interest.

After obtaining the ultrasound images, off-line measurements were performed. The tendon thickness was measured using the distance measurement software in the ultrasound machine. Measurements were taken from the inferior pole of patella vertically to the superior border of the patella tendon (Fig. 1A) using a trackball. The CSA of the patellar tendon (Fig. 1B) was measured by the tracing measurement software which allows the examiner to trace the outer margin of the tendon through the trackball, and the tracing measurement software was used to calculate the total area traced. The mean from the 3 measurements were used for statistical analysis.

Eleven healthy sedentary subjects were assessed twice with one week apart for test-retest reliability of patellar tendon thickness and CSA measurements. The inter-rater coefficient of correlation of tendon thickness and CSA were 0.94 (CV = 18.75%) and 0.98 (CV = 32.30%), respectively.

### Measurement of patellar tendon elastic modulus

B-mode was used to locate and align the patellar tendon longitudinally with the transducer. When a clear image of the patellar tendon was captured, the shear wave elastography mode was then activated. The transducer was stationed on the skin with light pressure on top of a generous amount of coupling gel, perpendicularly on the surface of the skin. The transducer was kept stationary for 8–12 seconds during the acquisition of the SSI map [16]. A total of 3 images were captured for the tendon on each knee for off-line analysis.

Off-line analysis was conducted and the procedures have been described in our recent paper [16]. The region of interest (ROI) was first defined by a rectangular box of 13.5 mm×12.5 mm (biggest size provided from the manufacturer) distal to the apex of the patella and with the patellar tendon located within its centre part. In the painful tendon, the circular quantification box (Q-Box) was centered where hypoechogenicity, disruption or fragmentation of collagen fiber, or focal sonolucent were observed. Similar to a previous study [11], the pathological lesions in our subjects were detected an average of 4.6 mm (ranged from 3–7 mm) distal to the apex of the patella (Fig.1C). The diameter of the Q-Box was determined by the width of the tendon. In the non-painful tendon, the Q-Box was centered at the proximal part of the patellar tendon with consistent images and at about 5 mm from the apex of the patella. Young's modulus (E) was estimated by the SSI system based on the following equation. E = 3ρc^2^, where ρ is the density (constant and equal to 1000 kg/m^3^) and c is the velocity of the shear wave propagation based on the assumption that the tissue is isotropic. A higher Young's modulus indicates greater stiffness [20]. The mean and maximum values of Young's modulus within the Q-Box were computed and displayed in kPa at the right bottom corner of the computer screen. The mean tendon shear elastic modulus was calculated by dividing the Young's modulus generated from the system by 3 [31]. The SSI has excellent test-retest reliability on patellar tendon shear elastic modulus (ICC: 0.98; coefficient of variation: 29.53%) [17].

The ratio of mean shear elastic modulus, tendon thickness and CSA between the painful and non-painful knee was calculated as elastic ratio, thickness ratio and CSA ratio in athletes with unilateral PT.

### Clinical Evaluation

Pressure pain was measured by a hand-held algometer (manufactured by pdt, Rome, Italy). Pain was provoked through a rubber disc at the end of the algometer. The participant was positioned in supine lying with 30° of knee flexion on the couch. The most painful area on the proximal patellar tendon was determined by palpation and then a 10lb force was applied via the algometer onto this area. The intensity of pain being provoked was reported using a visual analogue scale (VAS) from 0 to 10, with 0 indicating no pain, and 10 indicating the worst pain during testing. The VAS scale is a reliable and valid scale in the evaluation of patients with anterior knee pain [32].

The VISA-P questionnaire is used to assess the severity of symptoms and functional ability in subjects with PT [33]. The questionnaire comprises 8 questions with 4 on self-perceived pain associated with a functional activity, 2 on the ability in performing functional activities, and 2 on the ability to play sport. Self-perceived pain or abilities are rated on a 10-point Likert scale with 0 being the worst pain or lowest ability and 10 as the least pain and highest ability. The total score of the questionnaire is 100 and the final score would quantify the functional level. The VISA-P questionnaire has been used for studies on PT in various athletic populations [2], [34].

### Statistical Analysis

Independent *t* tests were performed to compare the demographic data between athletes with and without PT. After the normality distributions were confirmed using the Shapiro-Wilk tests, paired *t*-tests were used to compare the outcome measures (thickness, CSA and shear elastic modulus of patellar tendon) between the dominant and non-dominant sides in the healthy athletes, and also the painful and non-painful sides in athletes with unilateral PT. Univariate analysis of covariance tests were used to compare between the affected side in athletes with PT and the dominant side of the controls with demographic factors that demonstrated significant group difference as covariates. Spearman's rank correlation tests were used to examine the thickness ratio, CSA ratio and elastic ratio with the pressure pain, individual and total scores of the VISA-P questionnaire. SPSS version 17.0 (SPSS Inc, Chicago, IL) was used to perform statistical analyses. A *p* value of <0.05 was considered as significant for each of the measurements.

## Results

### Participant's demographic data

Participants' age, height, weight, BMI and training intensity in the two groups are shown in Table 1. No significant differences were found between the two groups in age, height, weight, BMI and training intensity (*p*>0.05), but a medium trend was found in BMI between the 2 groups (*p* = 0.094; Cohen's *d* = 0.52).

**Table 1:** Demography comparison between athletes with and without unilateral PT.

Variables	Control group	PT group	*P* Value
	(n = 20)	(n = 13)	
Age, y	24.9±4.4	22.9±4.6	0.222
Weight, kg	73.4±7.9	76.2±6.3	0.280
Height, cm	181.7±6.0	180.0±5.7	0.425
BMI, kg/m^2^	22.2±2.1	23.6±2.4	0.094
Sport-specific training, h/wk	7.5±3.7	5.4±2.5	0.102
Injury duration, y		1.7±1.6	
Dominate/non-dominate side (painful side)		8/5	

Values shown as mean± standard deviation; PT = patellar tendinopathy; BMI = body mass index.

### Side-to-side differences on thickness, CSA and shear elastic modulus

In athletes with unilateral PT, B-mode ultrasound measurements revealed significant differences between the painful and non-painful sides in patellar tendon thickness (*p* = 0.001) and CSA (*p* = 0.002) (Table 2). On average, the painful tendons were thickened by 33.3% and enlarged by 12.5% in CSA than the non-painful side. The shear elastic modulus in the painful side (mean: 43.6 kPa) was significantly higher than the non-painful side (mean: 25.8 kPa) by 40.8% (*p* = 0.008).

**Table 2:** Comparisons of the shear elastic modulus, thickness and CSA between painful and non-painful sides in athletes with unilateral PT.

Variables	Painful side	Non-painful side	*P* Value
	(n = 13)	(n = 13)	
Shear elastic modulus, kPa	43.6±17.9	25.8±10.6	0.008*
Thickness, mm	6.9±1.8	4.6±0.6	0.001*
CSA, cm^2^	1.7±0.4	1.4±0.3	0.002*

Values shown as mean± standard deviation; PT = patellar tendinopathy; CSA = cross sectional area. *P<0.05.

Side-to-side differences on the outcome measures were not observed in healthy athletes. There were no significant differences on the patellar tendon morphology and elastic properties between the dominant and non-dominant sides (*p*>0.05) despite a trend of increase in patellar tendon thickness in the dominant than the non-dominant leg (*p* = 0.095) (Table 3). The mean tendon thickness, CSA and shear elastic modulus were 5.6 mm, 1.4 cm^2^ and 27.5 kPa in the dominant leg; 5.3 mm, 1.4 cm^2^, and 27.9 kPa in the non-dominant leg.

**Table 3:** Side-to-side comparisons of the shear elastic modulus, thickness and CSA in healthy athletes.

Variables	Dominant side	Non-dominant side	*P* Value
	(n = 20)	(n = 20)	
Shear elastic modulus, kPa	27.5±11.3	27.9±8.4	0.868
Thickness, mm	5.6±1.2	5.3±1.0	0.095
CSA, cm^2^	1.4±0.3	1.4±0.3	0.917

Values shown as mean± standard deviation; CSA = cross sectional area.

### Comparison of shear elastic modulus, thickness and CSA between healthy athletes and athletes with unilateral PT

Group differences were observed on the patellar tendon morphology and elastic properties between athletes with PT and without PT. The mean group differences on the patellar tendon thickness and CSA were 1.3 mm and 0.3 cm^2^, respectively (Table 4). The shear elastic modulus was increased from 27.5 kPa to 43.6 kPa (by 36.9%, *p = *0.003) in the painful tendon among athletes with PT when compared with the controls.

**Table 4:** Comparisons of the shear elastic modulus, thickness and CSA of the patellar tendon on the painful side of athletes with unilateral PT and dominant side of the healthy athletes.

Variables	Control group	PT group	*P* Value
	(n = 20)	(n = 13)	
Shear elastic modulus, kPa	27.5±11.3	43.6±17.9	0.003*
Thickness, mm	5.6±1.2	6.9±1.8	0.019*
CSA, cm^2^	1.4±0.3	1.7±0.4	0.032*

Values shown as mean ± standard deviation; PT =  patellar tendinopathy; CSA =  cross sectional area.*P<0.05.

### Relationships between changes in tendon morphology, elastic properties, pressure pain and dysfunctions

**Figure 2:**
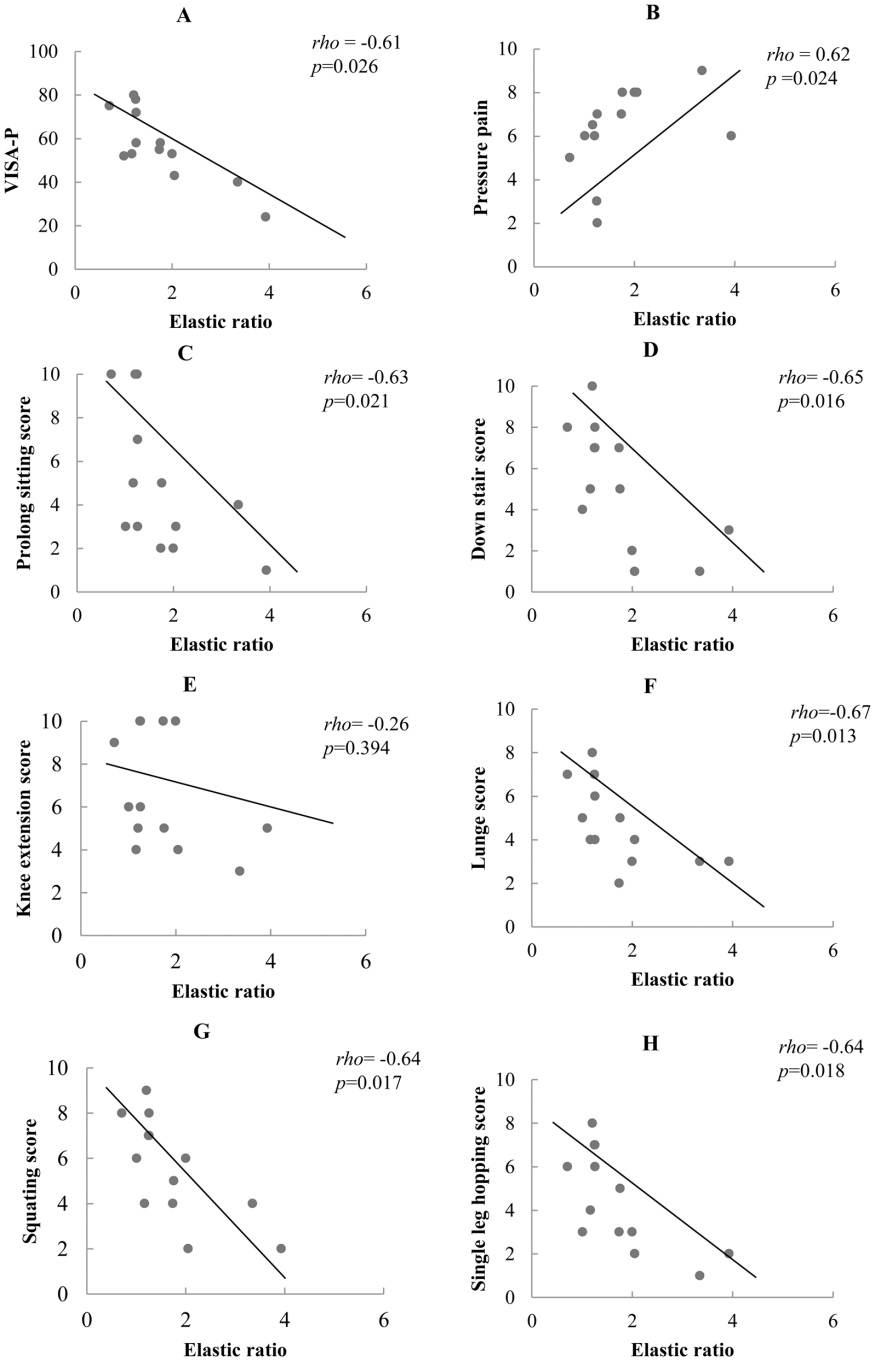
Correlations between elastic ratio and clinical variables. (Pressure pain, individual and total score from VISA-P, Victorian Institute of Sport Assessment- Patella).

Table 5 shows the relationships between changes in tendon properties, pressure pain and dysfunctions. Significant negative correlation was found between elastic ratio and VISA-P scores (*rho*  = −0.61; *p* = 0.026) (Fig. 2A). Significant positive correlation was found between elastic ratio and pressure pain (*rho*  = 0.62, *p* = 0.024) (Fig. 2B) and negative relationships were established between elastic ratio and self-perceived pain based on the sub-scores from the VISA-P questionnaire (*rho* ranged from −0.63 to −0.67; *p*<0.05) (Fig. 2C, 2D, 2F, 2G, 2H) except for the knee extension (*rho* = −0.26, *p* = 0.394) (Fig. 2E). A higher ratio, greater differences between the painful and non-painful tendon, was associated with greater intensity of pain with pressure and when performing forward lunge, going down stairs and single leg hopping; as well as greater dysfunctions. Similar relationships could not be detected with thickness ratio and CSA ratio.

**Table 5:** Spearman's rank correlations between the ratio of tendon thickness, CSA and shear elastic modulus of the painful and non-painful side with intensity of pressure pain, individuals and total VISA-P scores.

		Morphology		Elastic properties
Pain		Thickness ratio	CSA ratio	Elastic ratio
	Pressure pain	0.53	−0.04	0.62*
	Downstairs	−0.28	−0.22	−0.65*
	Knee extension	0.02	0.42	−0.26
	Single leg hopping	−0.30	−0.13	−0.64*
	Lunge	−0.11	−0.07	−0.67*
Ability				
	Prolong sitting	−0.46	−0.15	−0.63*
	Squatting	−0.14	−0.00	−0.64*
Dysfunction				
	VISA-p score	−0.25	−0.07	−0.61*

Abbreviations: CSA = cross sectional area; VISA-P =  Victorian Institute of Sports Assessment-patella Questionnaire; *P<0.05.

## Discussion

The present study revealed changes in patellar tendon morphology and elastic properties in athletes with unilateral PT lasting from 3 months to 6 years. The painful tendons were thicker and larger in size with an increase in stiffness when compared with the non-painful side and healthy control subjects. This study also established associations between elastic properties of patellar tendon and intensity of pressure- and activity-related pain, as well as dysfunctions in basketball and volleyball players with unilateral PT.

Morphological changes such as thickening and larger CSA in pathological tendons, have been reported [3], [5], [6] and the changes were about 22.7% in thickness [5] and 15.8% in CSA [6] in the proximal part of the patellar tendon. Cook et al. [7] even advocates the use of ultrasound imaging together with clinical examination in making diagnosis of tendinopathy. In this study, patients were confirmed to have PT based on both clinical examination and ultrasound imaging, it is therefore not surprising to find differences on the size and CSA of the pathological tendons from the unaffected side or the patellar tendon of the healthy controls. In the present study, the tendon size was increased by 35.7% and the CSA was enlarged by 21.4% in the pathological tendons. These changes could be explained by an increase in ground substance [35], collagen fiber disorganization [4], and hypercellularity [36].

One of the main findings from this study was the change in tendon elastic properties in athletes with unilateral PT using supersonic shear imaging technique. The painful tendons were stiffer than the non-painful side as well as the dominant side of the healthy controls. The measurements were made on the proximal portion of the tendon where pain was elicited on palpation and perceived during functional activities of the lower limb. Patellar tendon elastic properties were previously quantified by ultrasonography with dynamometry. Kongsgaard et al. [9] did not find any difference in the tendon stiffness between 9 healthy individuals and 8 subjects with patellar tendinopathy. A later study by Couppé et al. [10] also reported no difference on the tendon stiffness between the painful and non-painful sides of 7 badminton players with unilateral PT and also compared with 9 control subjects. Based on a relatively larger sample, Helland et al. [8] found significantly lower tendon stiffness (by 21.4%) in the patellar tendon in 13 volleyball players with PT when compared with 15 controls. In these studies, elastic properties of the whole tendon were measured so as to unveil the entire physical properties of the tendon. We were more interested to investigate regional-specific changes around the pathological region, where pain is normally elicited.

Similar to our study, regional-specific increase in tendon stiffness was reported in subjects with chronic pain in the Achilles tendons by Sconfienza et al [12]. The stiffness of Achilles tendons was assessed by ultrasound sonoelastography at the myotendinous junction, tendon body and calcaneal enthesis. Loss of elasticity was detected in the tendon body but not in the myotendinous junction or calcaneal enthesis when compared with control. On the contrary, De Zordo reported decrease in tendon stiffness at the common extensor origin in patients with lateral epicondylitis [13]. More study is required to examine whether tendinopathy associated changes in elastic properties would be different in the lower and upper limb tendons.

The study from Sconfienza et al. [12] observed lower elastic values in areas with fragmentation and loss of fibrillar texture. In our study, the tendon shear elastic modulus was measured at the proximal part of the patellar tendon in area of hypoechoic or fragmentation signals based on ultrasound imaging. On the contrary, lower tendon stiffness was detected in subjects with acute Achilles tendon rupture [18] and in partial tendon tears in a porcine model [19]. Chen et al. [18] commented that the shearwave images could not be registered in areas with hematoma. The elastic value was dramatically reduced to 0 thus lowering the mean value. Furthermore, changes that occur in tendons with tendinopathy but not in tendons with acute rupture include transition of collagen fibers between Type I and Type III [23], [37]; disorganization of collagen fibers [4], increase in collagen cross-links [24] and formation of scar tissues [38]. These changes would likely increase tissue elastic modulus. Biochemical and histological tests are suggested to assess changes on collagen fibers, extracellular matrix and tendon elastic modulus.

The patellar tendon is short and thick where considerable force is transmitted. An inextensible tendon would have the most efficient force transmission. This tendon also serves other important functions such as energy storage/release upon loading and unloading and protection from muscle fiber injury [39]. To serve these functions, the patellar tendon exhibits spring-like characteristic, due to the presence of elastic components. Increased tendon stiffness in athletes with PT might be suitable for rapid and effective force transmission but could affect its function as mechanical buffer and elastic saving for economy of motion.

Besides measuring tendon elastic properties at rest, we also correlated the resting tendon elastic properties with pain and dysfunctions. Significant correlations were found between pain and dysfunctions with modulation on the tendon elastic properties but not with tendon morphology (thickness and CSA). Patients with PT are characterized with pain at the proximal patellar tendon associated with activities that load the tendon [3]. The present findings provide evidence that a greater increase in stiffness of the painful tendon, i.e. greater ratio on the shear elastic modulus between the painful and non-painful side, are associated with the self-perceived pain with local pressure and during tendon-loading activities. Most importantly, the dysfunctions as reflected from the VISA-P scores are related with the modulation in the tendon elastic properties. Tendon pain is closely linked to loading while excessive energy storage and release (over stretch) to the tendon would most commonly provoke pain [40]. We could not establish a relationship between the change in tendon morphology with pain or dysfunction. Malliaras et al. [25] found that hypoechoic area and diffuse thickening in patellar tendon were more likely to be painful. However, Warden et al. [41] and the present study could not establish a relationship between the CSA of patellar tendon and the VISA-P scores in athletes with unilateral PT.

Interestingly, there was not a significant side-to-side difference in the shear elastic modulus, thickness and CSA of patellar tendon between the dominant and non-dominant sides among healthy athletes. Such observations are partially in agreement with the findings from Couppé et al [42]. The authors found similar shear elastic modulus between the dominant and non-dominant legs. The thickness and CSA of patellar tendon, however, were found to be significantly thicker and larger in the leading leg in fencers and badminton players when compared with the other leg. A proposed explanation for this finding is the unilateral/asymmetrical training in these athletes. In our study, we recruited athletes in volleyball, basketball and handball that are generally regarded as bilateral sports.

### Limitations of the present study

The prevalence of PT is reported to be between 30% and 45% in the jumping athletes [2], but many of them suffered from bilateral PT. During the study period, 13 subjects with unilateral PT were recruited. Despite this small sample size, statistical significant difference was established on patellar tendon shear elastic modulus and thickness between athletes with and without PT. We also detected correlations between the tendon elastic modulus and the intensity of pain and functional scores. Such findings illustrated influence of tendon stiffness on pain and function. However, further study with larger number of subjects and in female athletes are suggested to support the present findings. Also, we conducted evaluation of tendon elastic properties on the proximal patellar tendon that does not represent the entire tendon. This is valid because the pathological changes in patellar tendon occurred mostly in the inferior pole of patella or proximal part of the tendon [6]. However, further study measuring tendon elastic modulus at different portions of the tendon would provide information on whether changes are isolated at the pathological region. In addition, ultrasound imaging was used to determine pathological lesions that had not been verified with histological tests. The present study was a cross-sectional study, we could not determine whether a stiffer proximal patellar tendon was the cause or consequence of the PT. Finally, only male athletes were recruited in this study, thus the findings from this study may not be generalized to female basketball, handball and volleyball players.

## Conclusions

The present study revealed changes in both morphology and elastic properties at the painful part of patellar tendon in athletes with unilateral PT. The affected tendons are stiffer, thicker and have larger cross-sectional area than the non-painful side and the tendon of healthy controls. In addition, the ratio of the painful and non-painful tendon elastic properties is associated with the intensity of pressure pain and VISA-p scores in athletes with unilateral PT.
